# Non-O1/Non-O139 Vibrio cholerae Bacteremia Presenting as Spontaneous Bacterial Peritonitis in Decompensated Cirrhosis: A Case Report

**DOI:** 10.7759/cureus.109579

**Published:** 2026-05-25

**Authors:** Karthika K.S., Rugma R., Dijo Darjees, Alfa A Siji, Radha Sugumaran

**Affiliations:** 1 Department of Microbiology, Sree Gokulam Medical College and Research Foundation, Thiruvananthapuram, IND; 2 Department of Microbiology, Jawaharlal Institute of Postgraduate Medical Education and Research, Puducherry, IND

**Keywords:** ascites, bloodstream infection, case report, cholera toxin negative, immunocompromised host, liver cirrhosis, liver decompensation, nono1/non-o139, novc bacteremia, spontaneous bacterial peritonitis

## Abstract

Non-O1/non-O139 *Vibrio cholerae* is an emerging invasive pathogen in immunocompromised individuals, particularly those with chronic liver disease, although it is more commonly associated with mild gastrointestinal illness. A 56-year-old male with decompensated cirrhosis presented with fever, abdominal distension, and abdominal pain, suggestive of spontaneous bacterial peritonitis (SBP). Ascitic fluid analysis supported SBP; however, ascitic fluid culture remained sterile. Blood culture flagged positive within 10 hours and revealed curved, Gram-negative bacilli identified as *V. cholerae*. Serotyping confirmed a non-O1/non-O139 strain, and polymerase chain reaction (PCR) assays for cholera toxin gene (*ctxA*) and toxin-coregulated pilus gene (*tcpA*) were negative. The isolate was susceptible to commonly tested antimicrobials, and the patient improved following treatment with piperacillin-tazobactam.

This report documents an unusual case of non-O1/non-O139 *V. cholerae* bacteremia presenting with SBP-like features in a cirrhotic patient and reviews the literature regarding the pathophysiology and clinical significance of this emerging pathogen in immunocompromised hosts, highlighting the importance of early recognition and prompt microbiological diagnosis for appropriate management.

## Introduction

*Vibrio cholerae* is a fermentative, Gram-negative, curved bacillus that is facultatively anaerobic and oxidase-positive, and it thrives in marine environments in close association with plankton [1]. These organisms are generally noninvasive, enterotoxigenic pathogens that cause acute diarrheal disease of varying severity. Although serogroups O1 and O139 are classically implicated in epidemic cholera because of their production of cholera toxin, non-O1/non-O139 strains are increasingly recognized as causes of invasive extraintestinal infections [2].

Bacteremia and septicemia caused by *Vibrio* species have been reported with increasing frequency over the past two decades [3,4]. Non-O1/non-O139 strains, which cannot be biochemically distinguished from *V. cholerae* O1, cause sporadic cases of diarrheal disease and invasive extraintestinal infections through virulence factors that differ from those of classical cholera toxin [5].

Bacteremia caused by non-O1/non-O139 *V. cholerae* predominantly occurs in individuals with underlying immunocompromised conditions, including cirrhosis, hematological malignancies, diabetes mellitus, end-stage renal disease, and previous gastrectomy [6]. The higher susceptibility in patients with cirrhosis is attributed to decreased serum bactericidal activity, impaired hepatic filtration, and high serum iron levels that promote bacterial proliferation [7].

Although non-O1/non-O139 *V. cholerae* infections are increasingly being recognized in immunocompromised individuals, reports describing bacteremia presenting with spontaneous bacterial peritonitis (SBP)-like features remain limited. This report highlights the clinical significance of this emerging pathogen and emphasizes the importance of early recognition and prompt microbiological diagnosis in high-risk patients.

## Case presentation

A 56-year-old man with a known history of decompensated cirrhosis (Child-Pugh class C with a score of 11, MELD-Na score of 15), portal hypertension, and alcohol use disorder (diagnosed in 2018) presented to the Medical Gastroenterology Outpatient Department in October 2025 with a four-day history of abdominal distension, abdominal pain, and fever. The patient reported being well four days before presentation, after which he developed progressive abdominal distension. This was followed by the onset of dull, aching, non-radiating abdominal pain that was unrelated to food intake. Concurrently, the patient developed high-grade, intermittent fever with chills and malaise. The patient denied vomiting, diarrhea, hematemesis, or melena. There was no history of exposure to sea or brackish water. There was also no history of cough, breathlessness, altered sensorium, or previous upper gastrointestinal bleeding.

On examination, the patient was conscious and oriented. The abdomen was distended with tense ascites. The temperature was 38.3°C, the pulse rate was 86 beats per minute, and the blood pressure was 130/80 mmHg. Routine blood investigations revealed a total leukocyte count of 7,780 cells/mm³ and a C-reactive protein level of 66.73 mg/L. Liver function tests showed abnormalities with elevated total bilirubin (4.17 mg%) and reduced serum albumin (2.6 g%). After diagnostic and therapeutic paracentesis (3.5 L) and after obtaining blood samples (two bottles) for culture, intravenous piperacillin-tazobactam (4.5 g every six hours) was initiated, along with supportive measures including albumin administration and fluid management.

Ascitic fluid analysis revealed a total leukocyte count of 5,790 cells/mm³ with 87.1% neutrophils (polymorphonuclear count >250 cells/mm³), consistent with SBP. Ascitic fluid protein was 0.8 g/dL, ascitic fluid albumin was 0.1 g%, and ascitic fluid glucose was 172 mg/dL (Table 1).

**Table 1:** Laboratory data upon the day of admission. TC: total count; CRP: C-reactive protein; DC: differential count

	Value	Normal range
Blood count		
TC	7780 cells/mm^3^	4000-11000
CRP	66.73 mg/L	<6 mg/L (negative)
Total bilirubin	4.17 mg%	0.2-1.0
Serum albumin	2.6 g%	3.5-5
Serum creatinine	1.12 mg%	0.7-1.3
Ascitic fluid		
TC	5790 cells/mm^3^	-
DC-neutrophils	87.1%	-
Protein	0.8 g/dL	-
Glucose	172 mg/dL	-
Albumin	0.1 g%	-

Ascitic fluid culture remained sterile after 48 hours of aerobic incubation. Both blood culture bottles flagged positive on BacT/ALERT after 10 hours of incubation.

**Figure 1:**
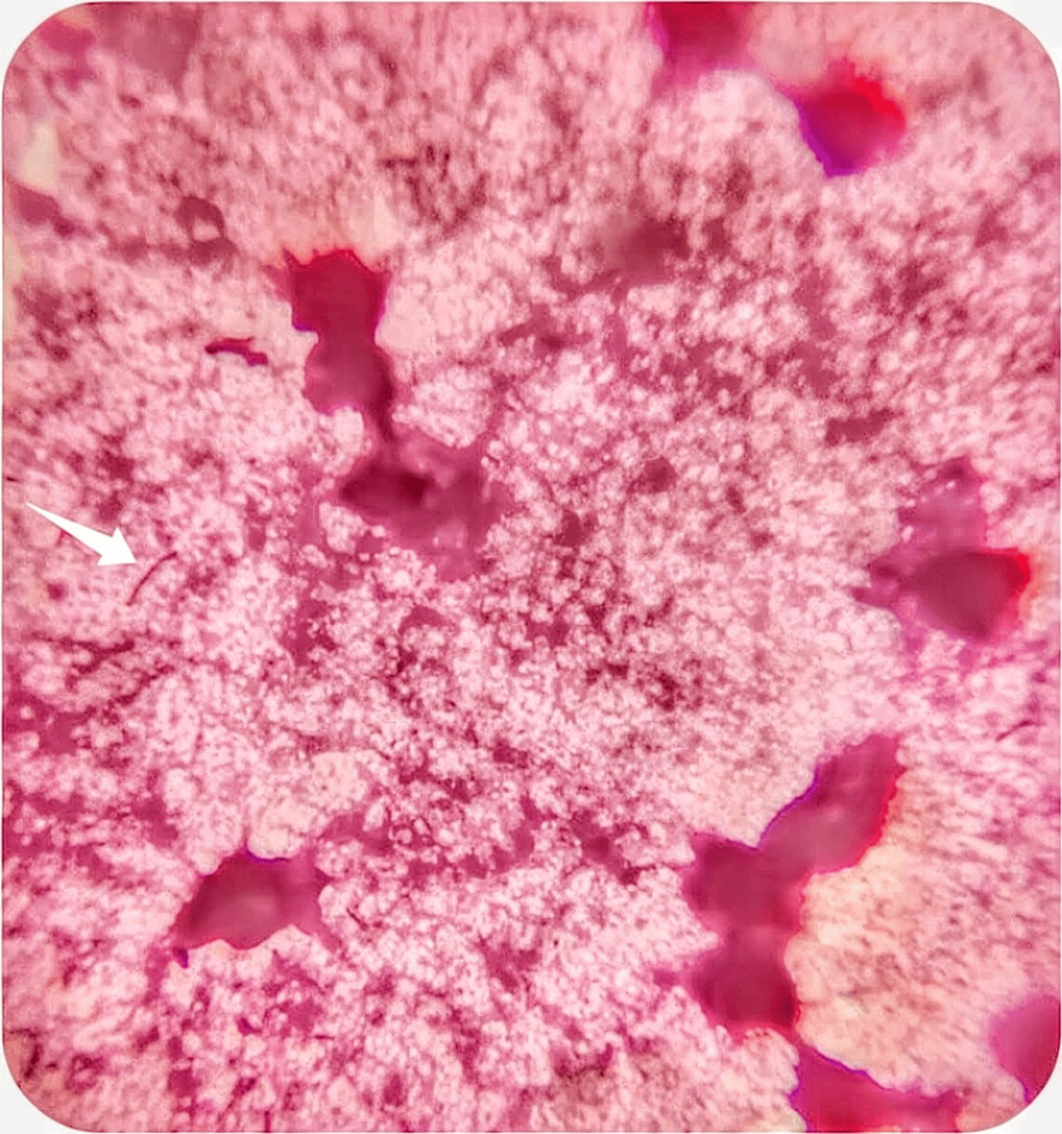
Gram’s stain of blood culture showing curved, Gram-negative bacilli (arrow).

Smears directly prepared from the blood samples revealed curved, Gram-negative bacilli (Figure 1).

**Figure 2:**
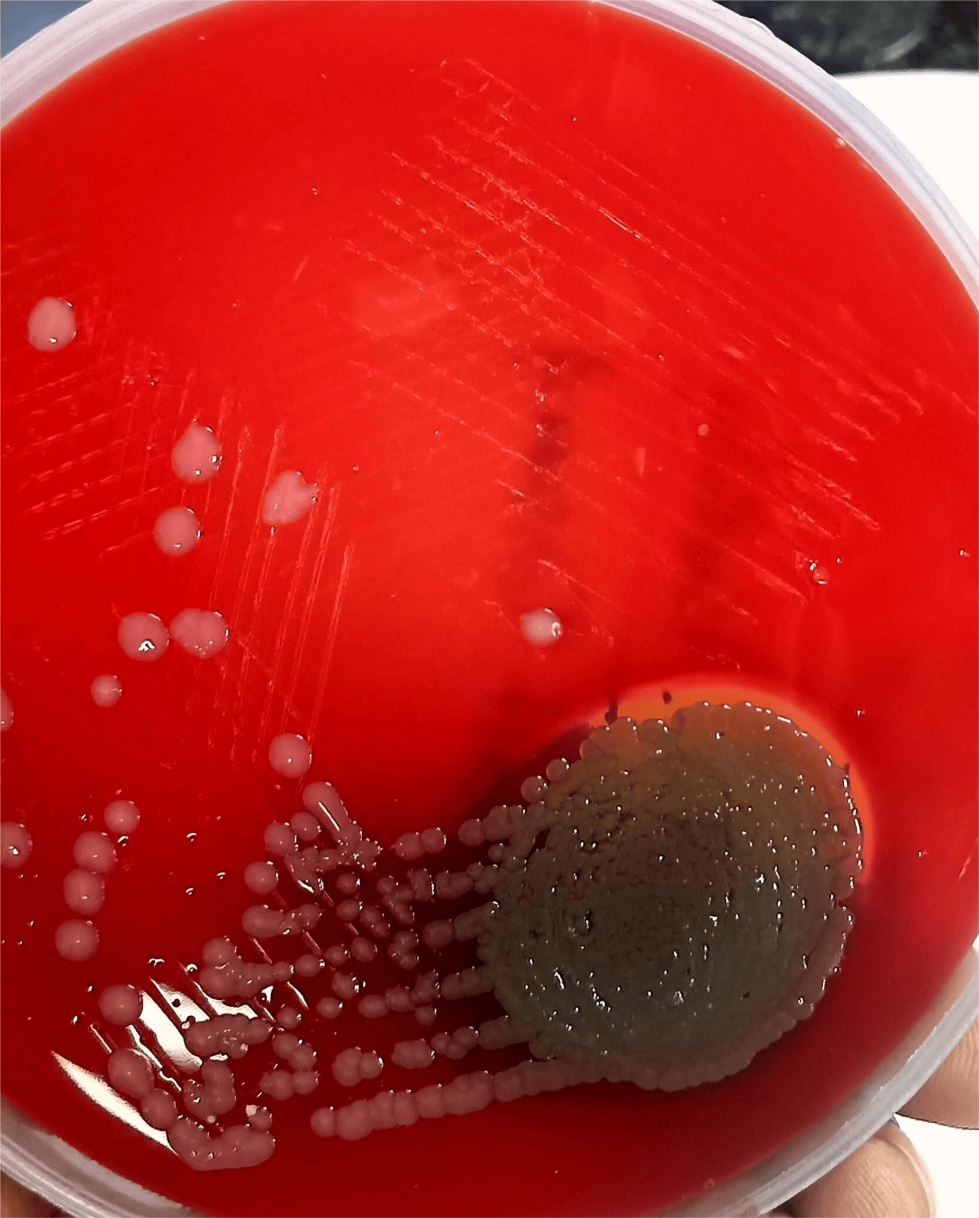
Blood agar showing smooth, convex, gray colonies.

**Figure 3:**
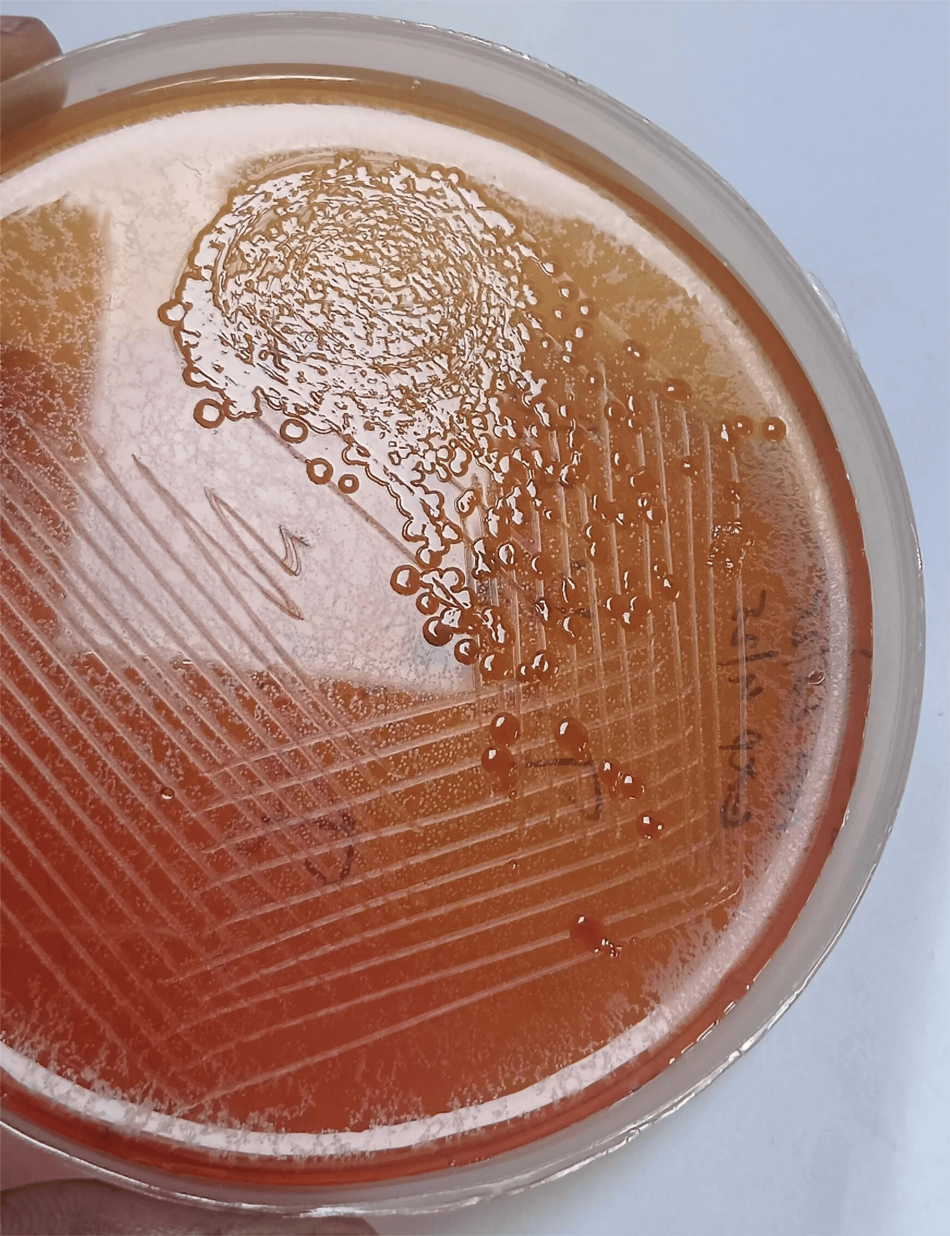
MacConkey agar showing non-lactose-fermenting colonies.

**Figure 4:**
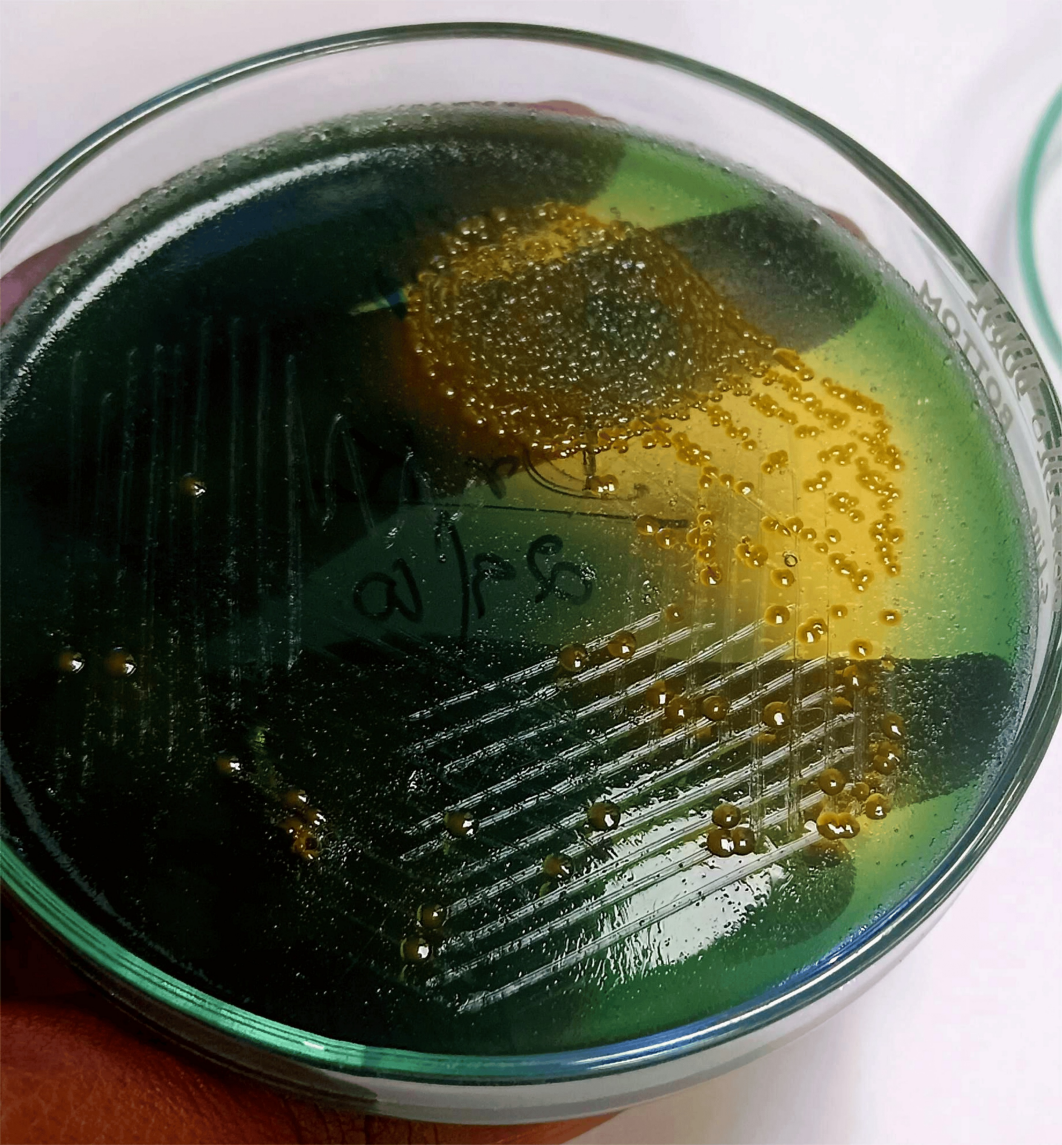
TCBS agar showing smooth yellow colonies. TCBS: thiosulfate-citrate-bile salts-sucrose

**Figure 5:**
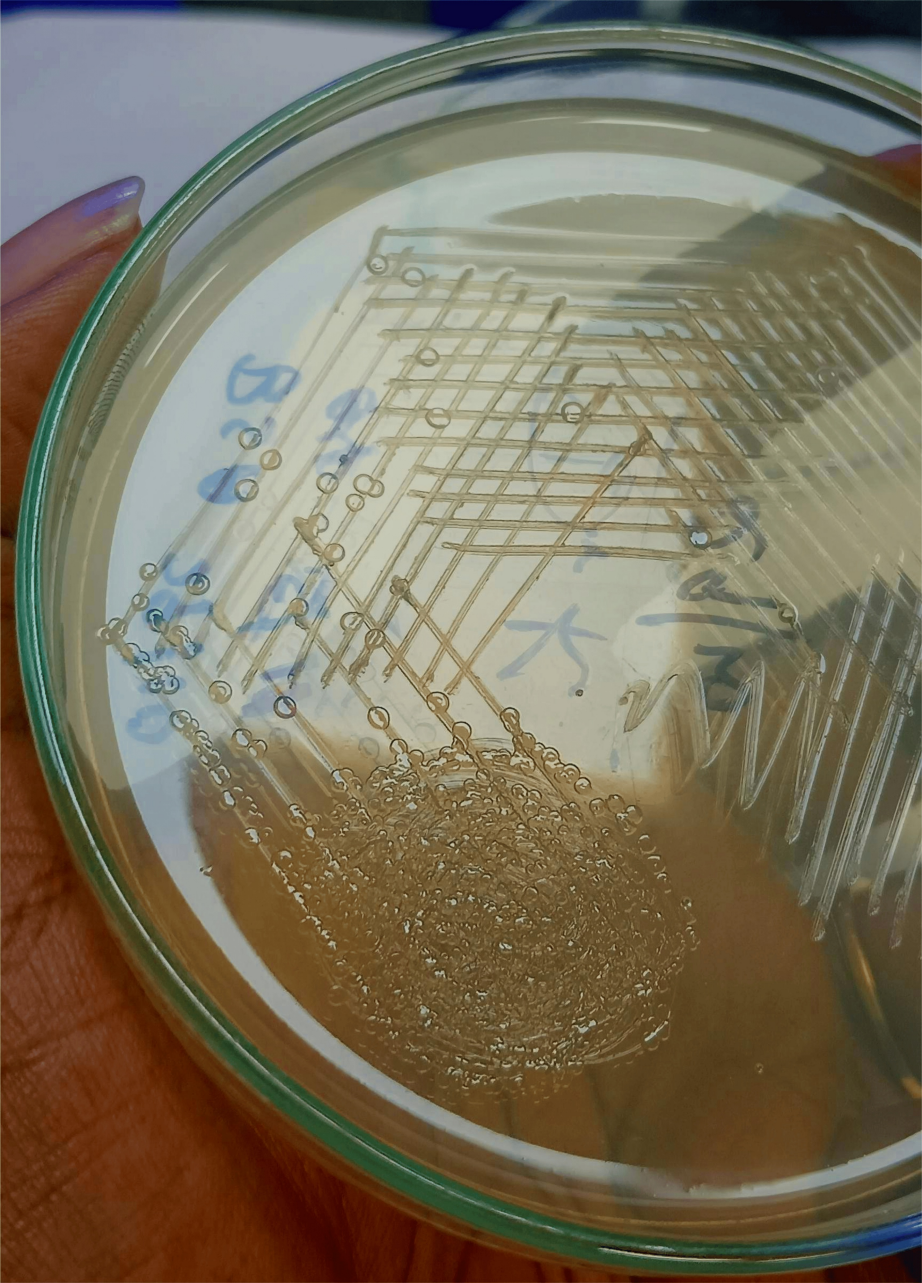
Bile salt agar showing characteristic “oil drop” colonies.

**Figure 6:**
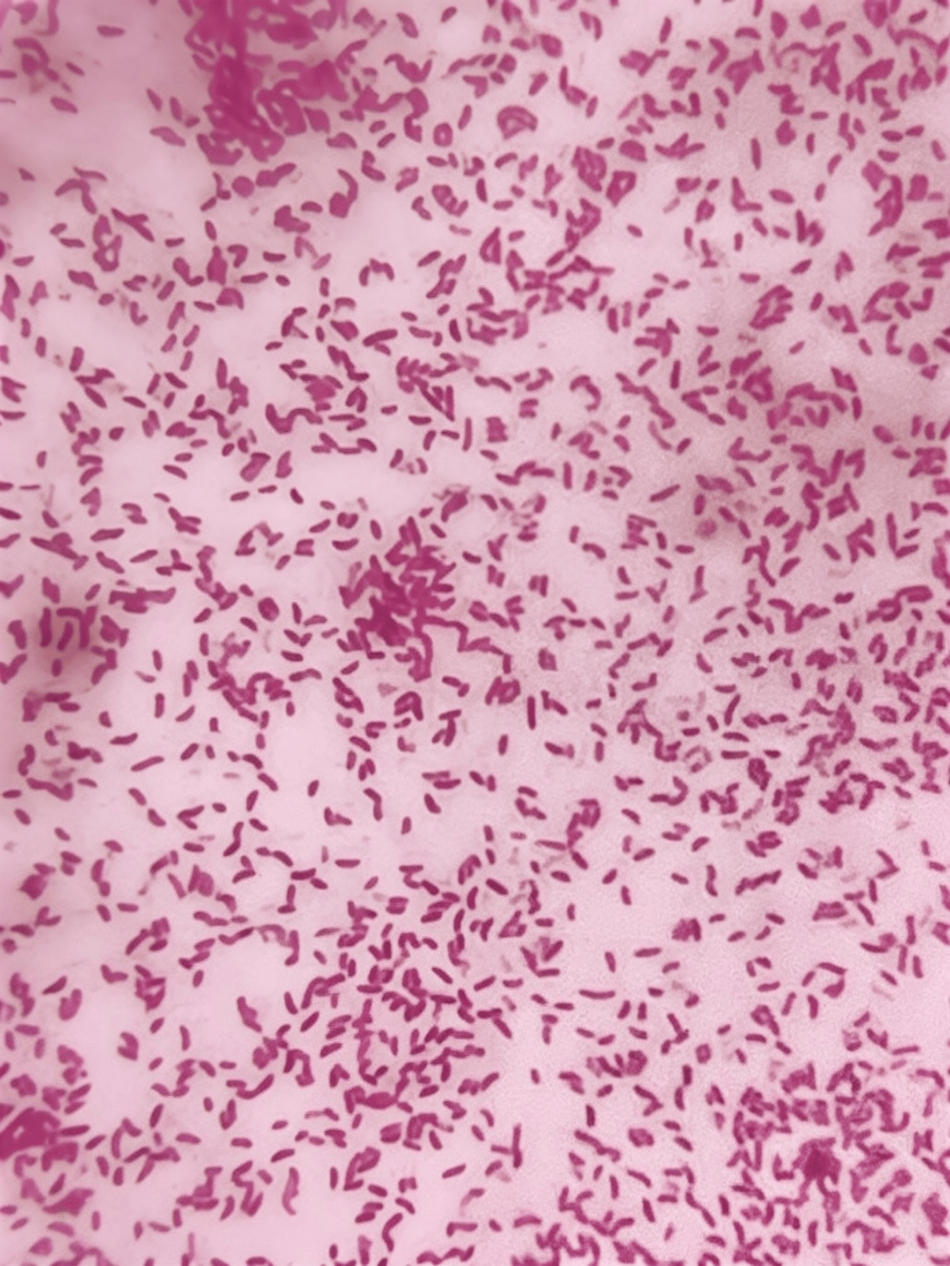
Colony smear showing curved, Gram-negative bacilli.

On subculture, smooth, convex, gray colonies were observed on blood agar (Figure 2), non-lactose-fermenting colonies on MacConkey agar (Figure 3), smooth yellow colonies on thiosulfate-citrate-bile salts-sucrose (TCBS) agar (Figure 4), and transparent, glistening “oil drop” colonies on bile salt agar (Figure 5). The isolate was oxidase-positive, catalase-positive, and motile on hanging drop preparation. Colony smear also showed curved, Gram-negative bacilli (Figure 6).

**Figure 7:**
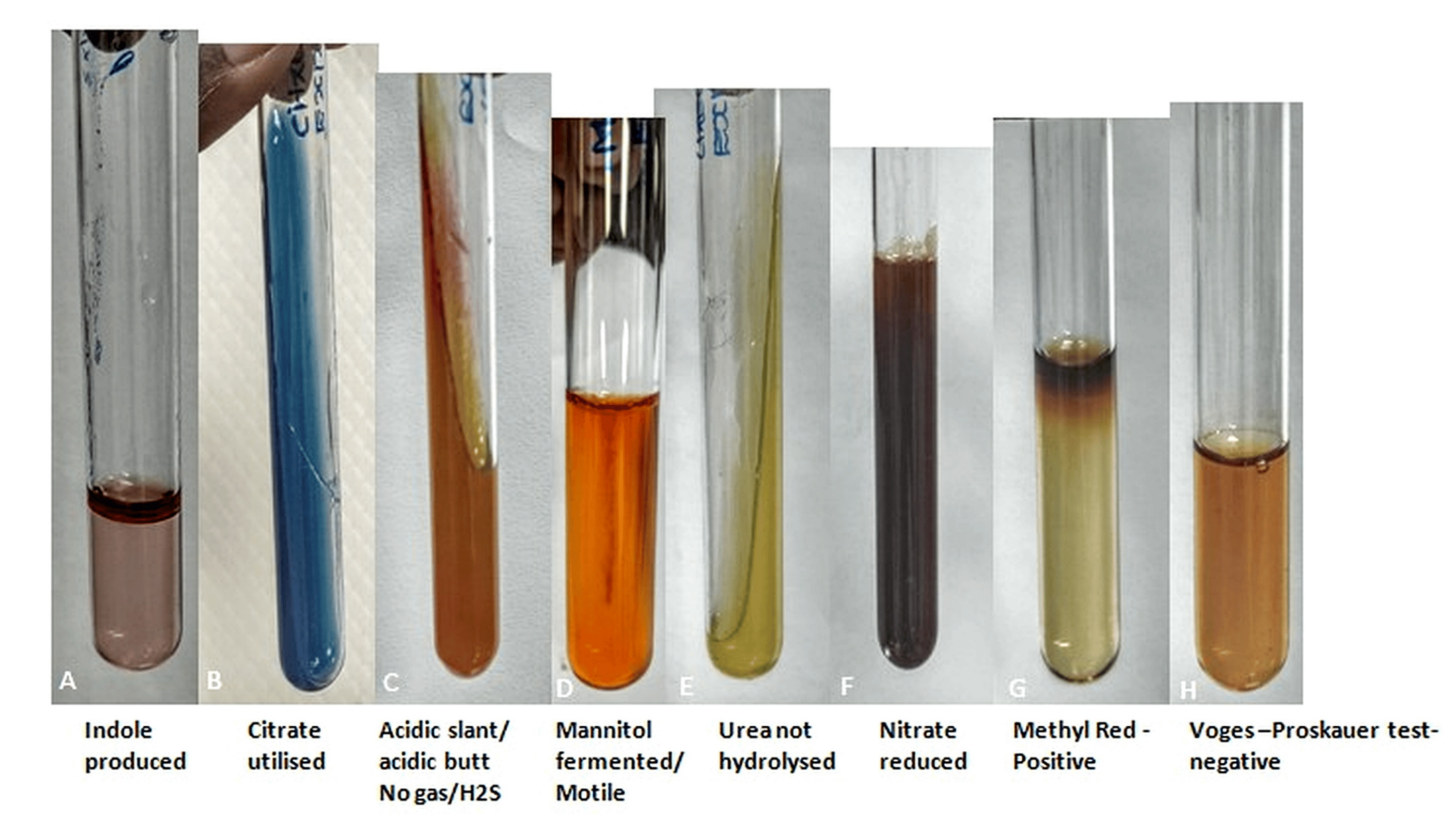
Biochemical test results. (A) Indole production; (B) citrate utilization; (C) TSI agar showing acidic slant/acidic butt without gas or hydrogen sulfide production; (D) mannitol fermentation and motility; (E) urea not hydrolyzed; (F) nitrate reduced to nitrite; (G) positive methyl red reaction; (H) negative Voges-Proskauer test. TSI: triple sugar iron

On triple sugar iron (TSI) agar, the organism produced an acidic slant and acidic butt without gas or hydrogen sulfide production. The isolate produced indole, reduced nitrate, and utilized citrate. The organism did not hydrolyze urea and was methyl red-positive and Voges-Proskauer negative (Figure 7).

**Figure 8:**
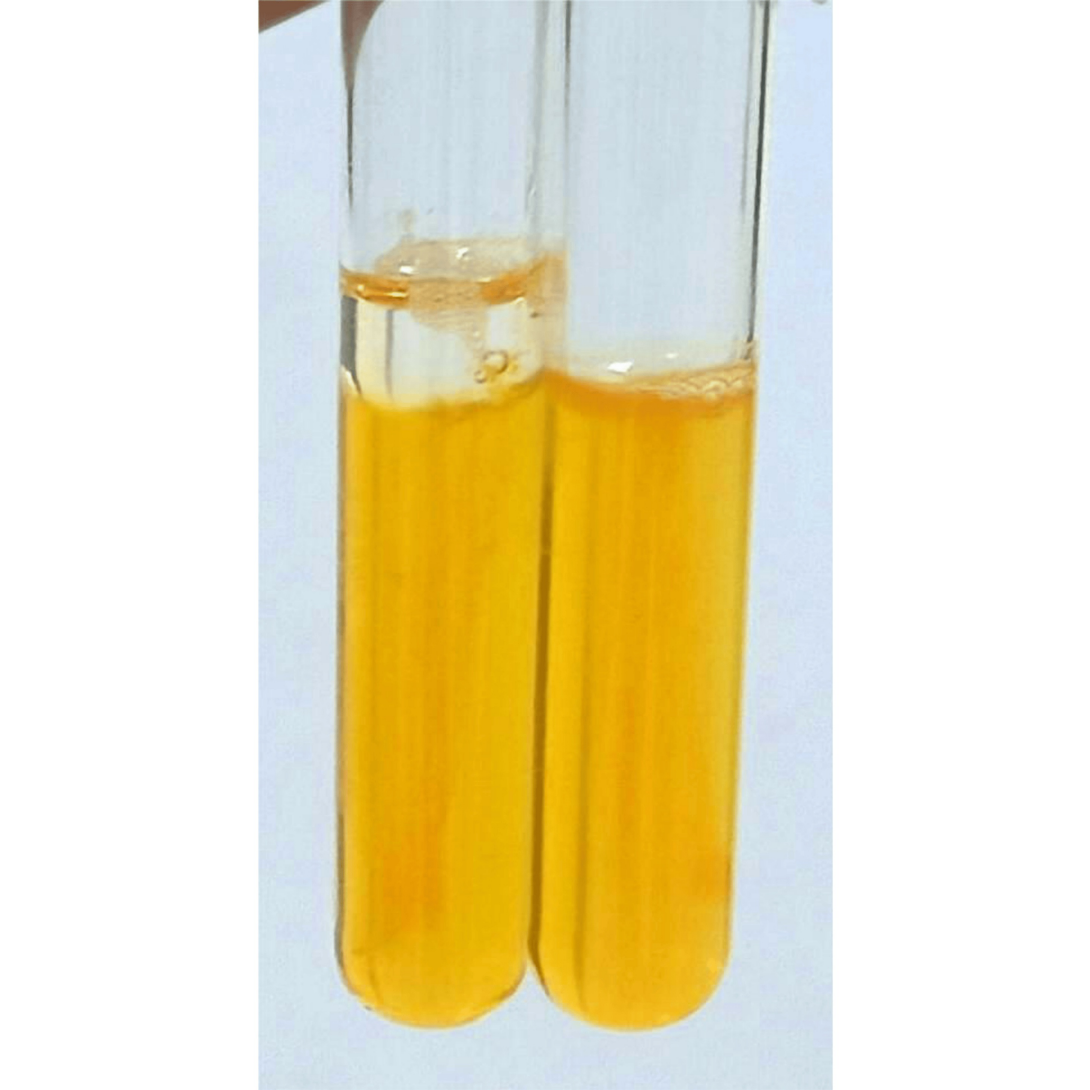
Oxidation-fermentation (OF) media. The yellow coloration in both tubes indicating oxidative and fermentative metabolism.

**Figure 9:**
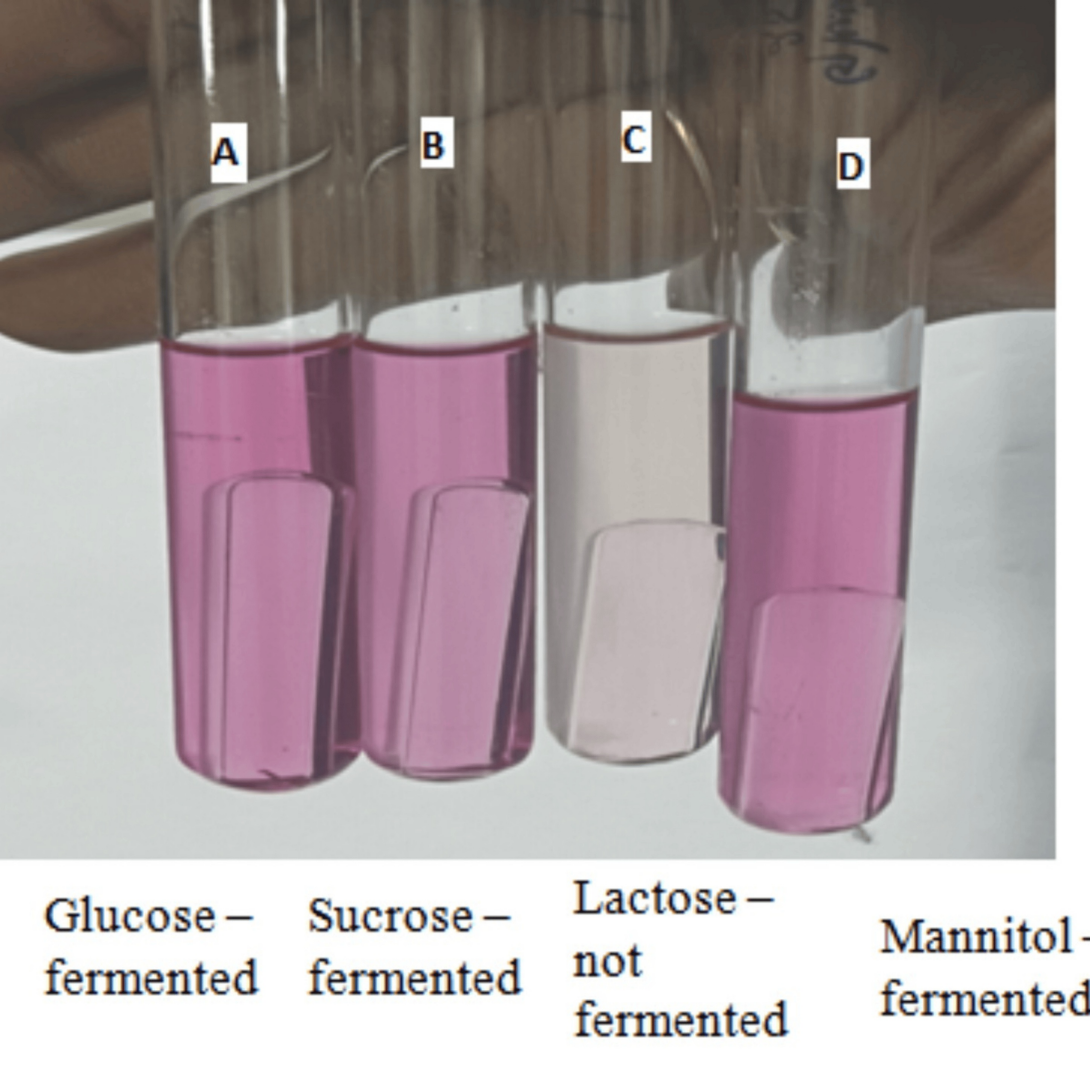
Sugar fermentation tests. (A) Glucose fermented; (B) sucrose fermented; (C) lactose not fermented; (D) mannitol fermented.

**Figure 10:**
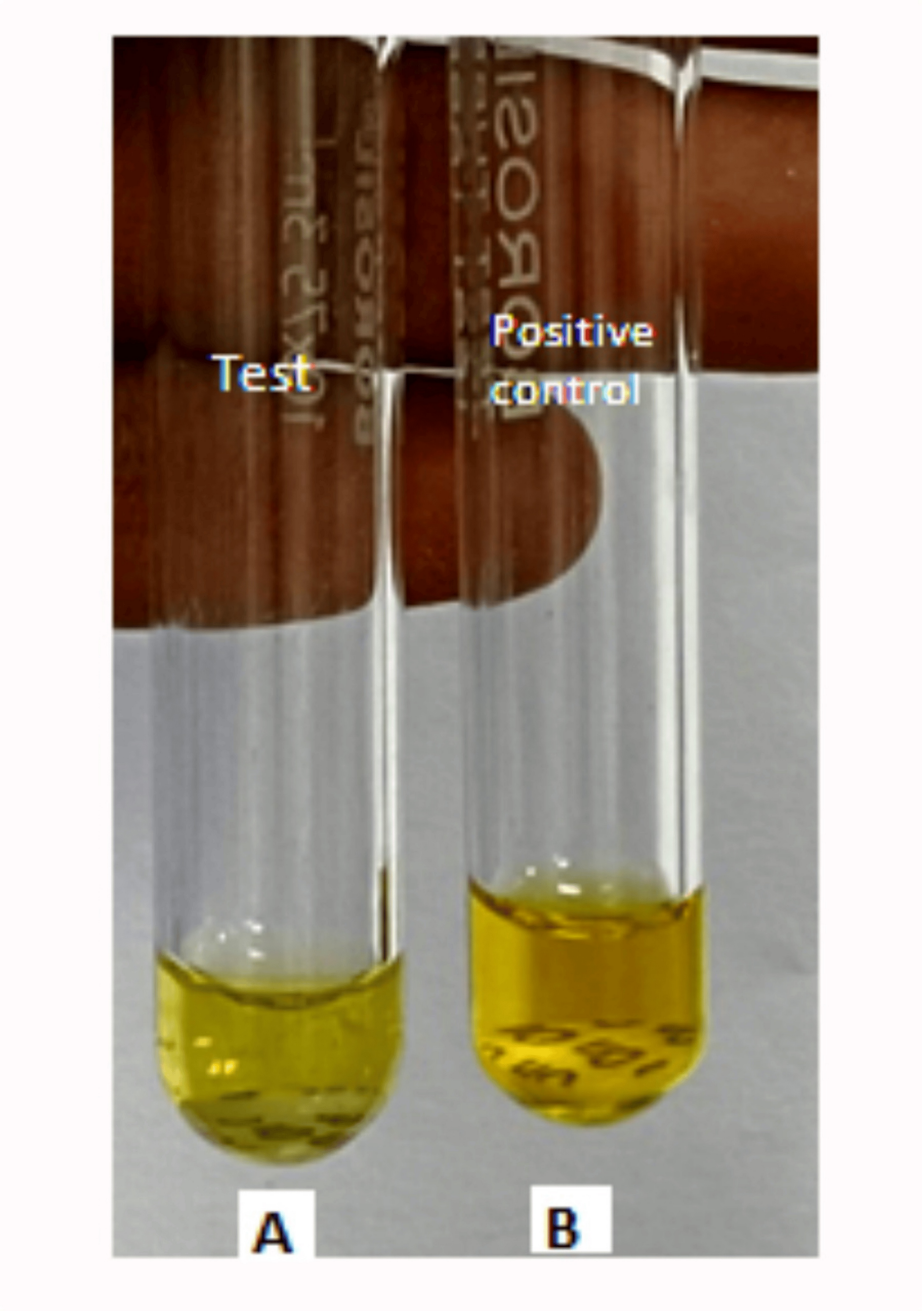
O-nitrophenyl-β-D-galactopyranoside (ONPG) test. (A) Test organism showing a positive reaction (yellow color) due to hydrolysis of o-nitrophenyl-β-D-galactopyranoside, indicating β-galactosidase activity in a late lactose-fermenting organism; (B) *Escherichia coli* (ATCC 25922) as positive control.

The isolate showed both oxidative and fermentative metabolism in oxidation-fermentation (OF) medium (Figure 8). It fermented glucose, sucrose, and mannitol without gas production, but did not ferment lactose (Figure 9). However, the o-nitrophenyl-β-D-galactopyranoside (ONPG) test was positive (Figure 10).

**Figure 11:**
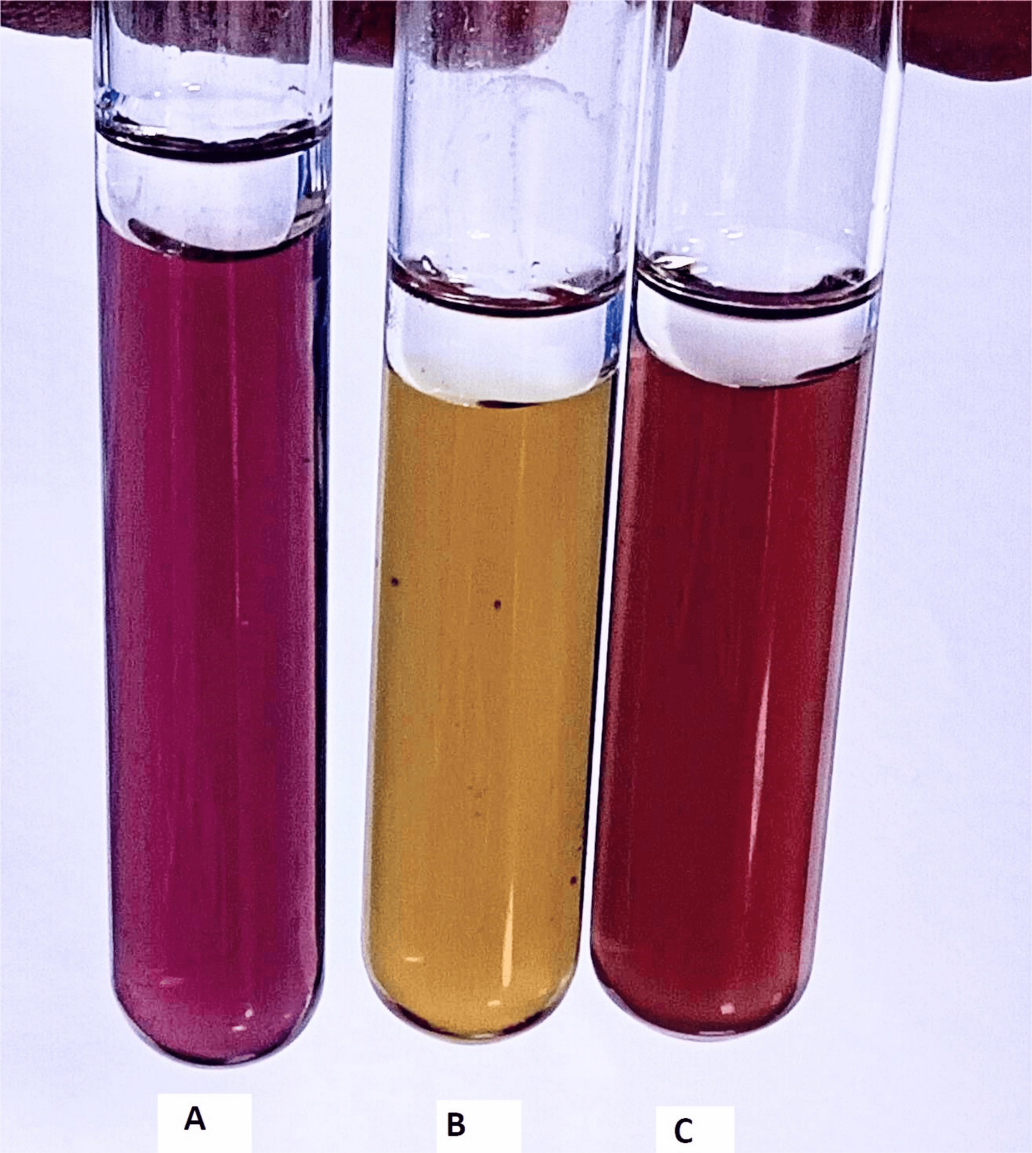
Amino acid metabolism tests. (A) Positive lysine decarboxylation; (B) negative arginine dihydrolase; (C) positive ornithine decarboxylation.

Lysine and ornithine decarboxylation tests were positive, whereas arginine dihydrolase was negative (Figure 11).

**Figure 12:**
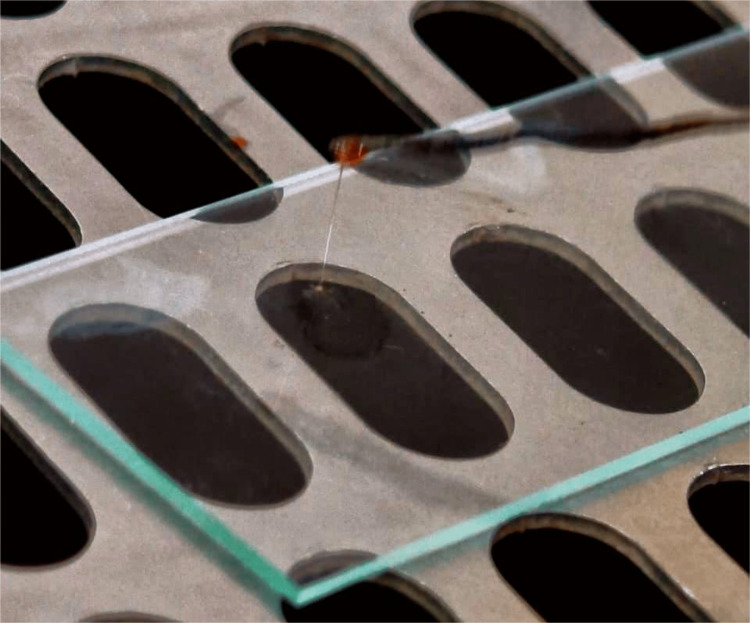
Positive string test. Formation of a viscous, mucoid string with 0.5% sodium deoxycholate.

**Figure 13:**
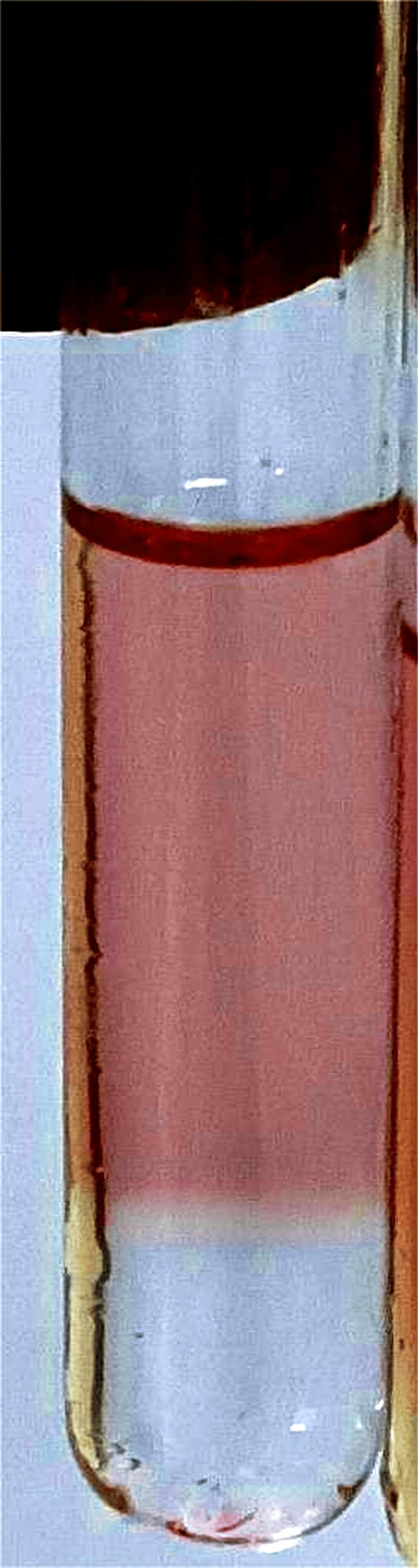
Positive cholera red reaction. Development of red coloration after the addition of concentrated sulfuric acid (H₂SO₄).

The isolate showed a positive string test (Figure 12) and a positive cholera red reaction (Figure 13).

The isolate did not agglutinate with polyvalent, Ogawa, or Inaba antisera. It was identified as *V. cholerae* using the VITEK 2 Compact system (bioMérieux, Marcy-l’Étoile, France) with the ID-GN card, with a 97% probability and a confidence level of “excellent identification.” Antimicrobial susceptibility testing was performed using the automated VITEK 2 Compact system and the Kirby-Bauer disk diffusion method on Mueller-Hinton agar. Results were interpreted in accordance with the Clinical and Laboratory Standards Institute M45 guidelines, Methods for Antimicrobial Dilution and Disk Susceptibility Testing of Infrequently Isolated or Fastidious Bacteria, 3rd edition [8]. The isolate was susceptible to ampicillin, amoxicillin-clavulanate, piperacillin-tazobactam, cefepime, cefotaxime, cefoxitin, ceftazidime, cefuroxime, imipenem, meropenem, azithromycin, amikacin, gentamicin, and tetracycline, and resistant to chloramphenicol, cotrimoxazole, and ciprofloxacin (Table 2).

**Table 2:** Antibiotic sensitivity in the blood culture. Using the Kirby-Bauer disk diffusion method and the VITEK 2 Compact system.

Antibiotic	Sensitivity
Ampicillin	Sensitive
Amoxicillin-clavulanate	Sensitive
Piperacillin-tazobactam	Sensitive
Cefepime	Sensitive
Cefotaxime	Sensitive
Cefoxitin	Sensitive
Ceftazidime	Sensitive
Cefuroxime	Sensitive
Imipenem	Sensitive
Meropenem	Sensitive
Azithromycin	Sensitive
Amikacin	Sensitive
Gentamicin	Sensitive
Tetracycline	Sensitive
Chloramphenicol	Resistant
Cotrimoxazole	Resistant
Ciprofloxacin	Resistant

The identification was further confirmed through matrix-assisted laser desorption/ionization time-of-flight mass spectrometry (MALDI-TOF MS) using the VITEK MS PRIME system, with a confidence level of 99.90%. Further characterization showed that the isolate was non-O1/non-O139 because it did not agglutinate with O1 or O139 antisera. Polymerase chain reaction (PCR) targeting the cholera toxin gene (*ctxA*) and toxin-coregulated pilus gene (*tcpA*) was negative.

The patient was continued on intravenous piperacillin-tazobactam and was discharged after four days of hospitalization following clinical improvement. Repeat blood cultures remained sterile after seven days of incubation.

## Discussion

This case describes an uncommon presentation of non-O1/non-O139 *V. cholerae* bacteremia that clinically mimicked SBP, with negative ascitic fluid cultures but positive blood cultures. While *V. cholerae* is classically associated with acute watery diarrhea caused by epidemic O1 and O139 serogroups, non-O1/non-O139 strains are increasingly recognized as causes of extraintestinal and invasive infections, particularly bloodstream infections in patients with underlying comorbidities [9-11].

In this patient, the clinical suspicion of SBP was high; however, the organism was not isolated from ascitic fluid despite documented bacteremia. This finding suggests that hematogenous dissemination after intestinal translocation may have occurred. Similar findings have been reported in patients with cirrhosis, in whom invasive *Vibrio* infections occur in the absence of diarrhea or positive ascitic fluid cultures. Possible explanations include a low bacterial burden in ascitic fluid, previous antimicrobial exposure, or transient or intermittent bacteremia that does not lead to sustained seeding of the peritoneal cavity. The absence of gastrointestinal symptoms in this case is also notable. Several reports of non-O1/non-O139 *V. cholerae* bacteremia describe similar presentations, and this absence of symptoms may delay clinical suspicion and diagnosis [3].

Accurate laboratory identification was crucial in this case. *V. cholerae* was isolated from paired BacT/ALERT FA Plus adult blood culture bottles, which flagged positive after approximately 10 hours of incubation. The culture broth was then subcultured onto solid media in accordance with standard laboratory operating procedures. The isolate was identified as *V. cholerae* by using the VITEK 2 automated identification system, MALDI-TOF mass spectrometry, and PCR-based confirmation. Serotyping confirmed that the isolate was non-O1/non-O139. PCR assays targeting the *ctxA* and the *tcpA* were negative. These findings support the nontoxigenic nature of the isolate and explain the absence of diarrheal manifestations. Differentiation from O1 and O139 serogroups is clinically important because non-O1/non-O139 strains lack classical cholera virulence factors and are more often associated with invasive disease rather than epidemic diarrhea [12]. Automated identification systems are generally reliable; however, confirmatory testing, serogrouping, and toxin gene detection remain necessary, particularly when isolates are recovered from sterile sites [13].

The epidemiology of non-O1/non-O139 *V. cholerae* bacteremia in India remains poorly defined because of the lack of structured surveillance and reliance on sporadic case reports. Most studies from India focus on diarrheal disease caused by *V. cholerae*, and invasive infections are likely underrecognized [14,15]. However, reports from different regions of India have documented extraintestinal infections and septicemia caused by non-O1/non-O139 strains, particularly in patients with chronic liver disease, diabetes mellitus, malignancy, and immunosuppression [16-18].

In South India, including Kerala, ecological and demographic factors may increase the risk of invasive *Vibrio* infections. The region’s extensive coastline, brackish water systems, monsoon-associated flooding, and high seafood consumption favor the environmental persistence of *Vibrio* species [19,20]. Environmental studies from coastal India have demonstrated the presence of non-O1/non-O139 *V. cholerae* in surface waters, supporting the likelihood of repeated human exposure [21]. Although published reports of bacteremia from Kerala are limited, the high prevalence of chronic liver disease in the state represents a significant host risk factor, consistent with global observations [11].

The pathogenic mechanisms underlying non-O1/non-O139 *V. cholerae* bacteremia remain incompletely understood. Unlike epidemic strains, these organisms possess alternative virulence determinants, including hemolysins, RTX toxins, and efficient iron acquisition systems, which facilitate survival and invasion in susceptible hosts [22]. In patients with chronic liver disease, high serum iron levels, portosystemic shunting, and impaired reticuloendothelial clearance further increase susceptibility to invasive *Vibrio* infections [4].

Antimicrobial susceptibility testing in this case demonstrated that the isolate was susceptible to ampicillin, azithromycin, and tetracycline, and resistant to chloramphenicol, cotrimoxazole, and ciprofloxacin. Susceptibility testing was performed using the disk diffusion method, and minimum inhibitory concentrations were determined using the VITEK 2 AST system. Results were interpreted according to the Clinical and Laboratory Standards Institute M45 guidelines, 3rd edition (2016). This resistance pattern is clinically significant, particularly fluoroquinolone resistance, because ciprofloxacin is commonly used to treat severe *Vibrio* infections. Increasing antimicrobial resistance among non-O1/non-O139 *V. cholerae* isolates has been reported in India, highlighting the importance of routine susceptibility testing to guide appropriate therapy [4,22-24].

There are no specific treatment guidelines for non-O1/non-O139 *V. cholerae* bloodstream infections, and management is extrapolated from the treatment of severe *Vibrio* infections. Early initiation of effective intravenous antimicrobial therapy, guided by susceptibility testing, remains the cornerstone of management [23]. Reported mortality rates for non-O1/non-O139 *V. cholerae* bacteremia range from 30% to 60%, particularly in patients with advanced liver disease, sepsis, or delayed initiation of appropriate therapy [25].

From a public health perspective, the isolation of non-O1/non-O139 *V. cholerae* from blood, although not associated with outbreaks, indicates environmental exposure and an expanding clinical spectrum of *V. cholerae* beyond classical cholera. For clinical microbiology laboratories, this case highlights the importance of maintaining vigilance for *Vibrio* species in blood cultures, particularly in high-risk patients. This case also demonstrates the value of advanced diagnostic methods, including MALDI-TOF, serogrouping, molecular confirmation, and susceptibility-guided therapy [12,13].

## Conclusions

This case highlights the expanding clinical spectrum of non-O1/non-O139 *V. cholerae* as an invasive pathogen in patients with advanced cirrhosis, presenting as bacteremia with SBP-like features in the absence of diarrhea. Sterile ascitic fluid culture with concurrent bloodstream infection suggests possible hematogenous dissemination, despite the paucibacillary nature of the ascitic fluid. The report underscores the importance of accurate microbiological identification and awareness of atypical presentations of non-O1/non-O139 *V. cholerae* infection in immunocompromised hosts.
